# An old diagnostic tool for new indications: inpatient Holter ECG for conditions other than syncope or stroke

**DOI:** 10.1038/s41598-023-39803-1

**Published:** 2023-08-02

**Authors:** Ophir Freund, Inbar Caspi, Idan Alcalay, Miriam R. Brezis, Shir Frydman, Gil Bornstein

**Affiliations:** Internal Medicine B, Tel-Aviv Sourasky Medical Center and Sackler Faculty of Medicine, Tel Aviv University, Wizman 6, Tel Aviv, Israel

**Keywords:** Arrhythmias, Diagnosis, Cardiovascular diseases

## Abstract

Holter electrocardiography (ECG) assists in the diagnosis of arrhythmias. Its use in the inpatient setting has been described solely for the evaluation of stroke and syncope. Our aim was to assess its diagnostic value for other conditions in the internal medicine department. We included all hospitalized patients between 2018 and 2021 in a tertiary referral center. The primary outcome was a diagnostic Holter recording a new arrhythmia that led to a change in treatment. Overall, 289 patients completed a 24-h inpatient Holter ECG for conditions other than syncope or stroke, with 39 (13%) diagnostic findings. The highest diagnostic value was found in patients admitted for pre-syncope (19%), palpitations (18%), and unexplained heart failure exacerbation/dyspnea (17%). A low diagnostic yield was found for the evaluation of chest pain (5%). Heart failure with preserved ejection fraction (adjusted OR 2.3, 95% CI 1.1–5.4, p = 0.04), and baseline ECG with either a bundle branch block (AOR 4.2, 95% CI 1.9–9.2, p < 0.01) or atrioventricular block (first or second degree, AOR 5, 95% CI 2.04–12.3, p < 0.01) were among the independent predictors for a diagnostic test. Inpatient Holter ECG monitoring may have value as a diagnostic tool for selected patients with conditions other than syncope or stroke.

## Introduction

Holter ECG monitors are simple devices that usually have three leads that continuously register electrocardiographic (ECG) recordings over 24–48 h^1^. Holter monitoring enables the quantification of the real burden of an arrhythmia, and the detection and recording of a rhythm disturbance^2^. Holter monitoring was traditionally used for the evaluation of an arrhythmia in the ambulatory setting^1,2^. The main cardiac monitoring method for inpatients is telemetry, which has evidence-based indications and guidelines^3–5^. However, telemetry use is limited by the lack of specialized teams to continuously watch and interpret the monitor’s recordings and by the ongoing need of telemetry beds for other indications, such as acute coronary syndrome or ischemic stroke^5–7^. These limitations led physicians to use 24-h Holter monitoring for hospitalized patients as well, mainly for the evaluation of arrhythmic syncope or cardiogenic stroke^7–10^. Inpatient Holter ECG is a relatively inexpensive test without any restrictions on patients' activities. On the other hand, using inpatient Holter monitoring for the above indications was previously shown to have a limited diagnostic yield^10,11^. Currently, there are no guidelines to set the standards for Holter use in the hospital setting for other conditions. The purpose of this analysis was to provide clinical evidence for the diagnostic value of Holter monitoring for cardiovascular conditions other than syncope and stroke in the setting of an internal medicine department.

## Methods

### Study design and participants

We conducted this observational cohort study at a tertiary referral hospital during a 3-year period (June 1, 2018, to June 1, 2021). Patients eligible for study participation were all those admitted to any of the nine internal medicine departments of the Tel Aviv Medical Center (TASMC) during the study period and completed inpatient 24-h Holter ECG monitoring during their hospitalization. The inclusion criteria were: (1) an indication for inpatient Holter ECG other than syncope or stroke, (2) availability of complete Holter findings, and (3) more than 6 h of monitor recording. The study was approved by the TASMC review board (0876-20-TLV) and conducted in accordance with the Declaration of Helsinki. Due to the nature of this retrospective study and the preserved anonymity of patients, a waiver of informed consent was waved by the TASMC institutional review board.

In our medical center, each patient that complete an inpatient Holter ECG monitoring is entered into an electronic database. We extracted all patients that underwent the test during our study period. We than reviewed all hospitalization progress notes and discharge summary of each patient to include patients based on our mentioned inclusion criteria. We selected and evaluated specific baseline characteristics based upon known risk factors for diagnostic Holter ECG monitoring from previous studies^12,13^. Hypertension, diabetes, and hyperlipidemia were included only if patients received a relevant treatment prior to admission. Ischemic heart disease and heart failure diagnoses were based on patient's medical records. Patients' ejection fraction was determined based on previous echocardiogram or those performed during the admission, with the more recent taken into account.

ECG recordings performed between hospital arrival and Holter monitoring were analyzed by one of the authors and compared with the interpretations of the treating physician (as recorded in patient's records). ECG findings were considered as being abnormal for analysis based upon former studies^14,15^, and included: any atrioventricular block (AVB), bradycardia (below 50 beats per minute), supraventricular tachycardia (SVT), bundle branch block (BBB), long QT interval (corrected QT interval above 460 ms) or signs of left ventricular hypertrophy (LVH, using the Sokolov-Lyon criteria). For the analysis we included all ECG abnormalities, regardless if they appear in previous ECGs or not.

All inpatient 24-h Holter results had been initially interpreted during the hospitalization by a cardiologist specialized in electrophysiology. The Holter test was termed diagnostic if it included an arrhythmia that was not present in previous ECG recordings and affected patient’s treatment. Two physicians from the research team (OF and IC) independently reviewed each case and decided if the Holter was considered diagnostic according to the definition above. In case of a disagreement, the opinion of a third reviewer (GB) was used for final decision.

### Indications for Holter monitoring and outcomes.

**Figure 1:**
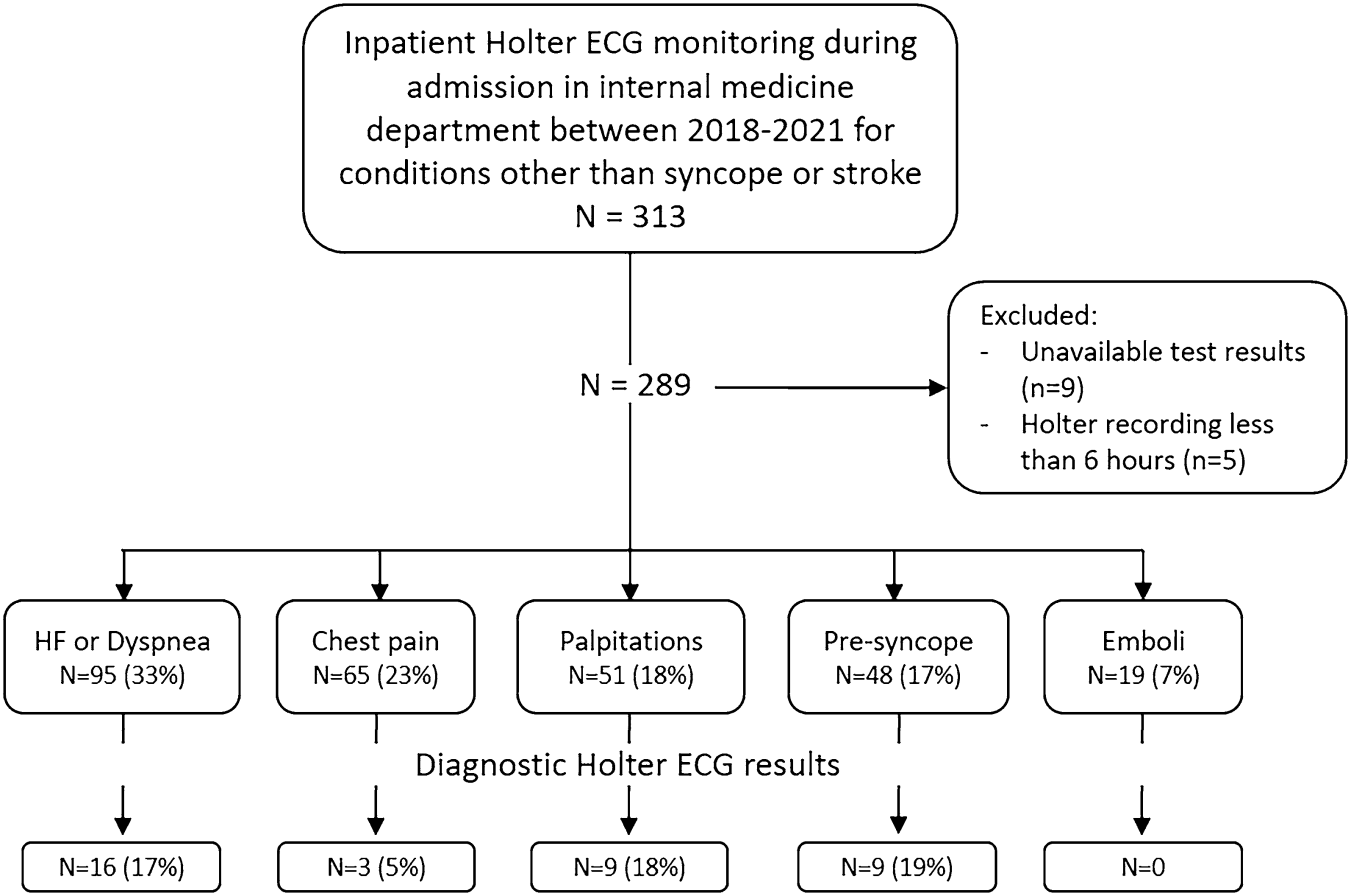
Inclusion process, main patient conditions and inpatient Holter ECG diagnostic yield.

The study population was divided into six groups based upon the main conditions that led to ordering the Holter monitoring (Fig. 1): (1) Unexplained heart failure (HF) exacerbation or dyspnea; (2) Palpitations; (3) Chest pain without evidence of acute coronary syndrome; (4) Pre-syncope (near fainting without loss of consciousness accompanied by weakness, blurry vision, or lightheadedness^16^); (5) Systemic emboli (evaluation of atrial fibrillation as a cause of systemic emboli); (6) Others.

The primary outcome of our study was the output of diagnostic Holter monitoring, meaning, a Holter report which led to a new diagnosis that was unknown upon admission and had therapeutic implications. We performed a descriptive analysis of the overall results of diagnostic Holter monitoring as well as for each of the main conditions, with evaluation of risk factors for the primary outcome by means of univariate and multivariate analyses. Secondary outcomes included time from admission to Holter monitoring and the influence of the Holter findings on treatment.

In an additional analysis, we divided the patients into two groups based on the main reason for inpatient Holter: (1) acute conditions with negative non-arrhythmic evaluations (unexplained dyspnea/HF exacerbation and chest pain), and (2) low suspicion for life-threatening arrhythmia with high-suspicion for arrhythmic etiology (palpitations, pre-syncope, and systemic emboli). We compared their baseline characteristics and risk factors for diagnostic Holter for a better implementation and personalization of our results.

### Statistical and cost analysis

Continuous variables were presented as mean ± standard deviation or median (interquartile range) for non-normally distributed variables. Categorical variables were presented as absolute number and percentage. To compare between categorical and continuous variables, Chi-square tests and independent t-tests were used, respectively. Mann–Whitney U tests were used to compare non-normally distributed continuous variables. Kolmogorov–Smirnov tests were used to assess for normal distribution. Independent risk factors for diagnostic Holter were evaluated by multivariate logistic regression models, and odds ratios (ORs) with 95% confidence intervals (CIs) were calculated. The analyses included independent variables/covariates that were statistically significant in the univariate analyses and age and sex as mandatory variables. A two-tailed p value of < 0.05 was considered significant for all analyses. All analyses were performed with the IBM SPSS statistics software version 27.0.

For cost analysis we calculated the direct costs of inpatient Holter monitoring from the Israeli Ministry of Health’s perspective based on the 2022 published price list. The cost in United States dollar (USD) of each Holter ECG test was $189.6 USD and each hospitalization day $787.5. We used a conservative estimation that Holter ECG resulted in one additional day of hospitalization (as a direct effect), meaning that undergoing Holter ECG monitoring during hospitalization cost $977.1 USD. To estimate the cost of achieving one diagnostic Holter ECG monitoring, we multiplied the cost of a single Holter with the frequency of diagnostic Holter for each indication.

### Ethics approval

The study was approved by the Tel-Aviv Sourasky medical center (TASMC) review board (0876-20-TLV) and conducted in accordance with the Declaration of Helsinki. Due to the nature of this retrospective study and the preserved anonymity of patients, a waiver of informed consent was waved by the TASMC institutional review board.

## Results

### Subject characteristics

During the study period, 289 patients completed an inpatient Holter ECG test for the evaluation of conditions other than stroke or syncope. Their baseline characteristics and clinical data are presented in Table 1. The cohort mean age was 75 ± 14 years and 48% were females. 36% had ischemic heart disease and 33% known atrial fibrillation. More than one-half (61%) of the patients had at least one abnormal finding in their baseline ECG with a median of 1 (0–1) abnormality. Unexplained HF or dyspnea (95 patients, 33%) and chest pain (65 patients, 23%) were the most prevalent conditions to indicate a Holter evaluation (Fig. 1). The median time between hospital admission to Holter monitoring was 2 (1–4) days.

**Table 1:** Characteristics of study cohort and comparison between patients with diagnostic and non-diagnostic Holter test.

Variable	Study cohort *n* = 289 (%)	Diagnostic Holter *n* = 39 (%)	Non-diagnostic Holter *n* = 250 (%)	*p*
Age, mean ± SD, year	75 ± 14	78 ± 14	75 ± 14	0.15
Female sex	140 (48)	17 (44)	123 (49)	0.51
Hypertension	209 (72)	36 (92)	173 (69)	< 0.01
Diabetes mellitus	102 (35)	13 (33)	89 (36)	0.78
Hyperlipidemia	171 (59)	18 (67)	153 (58)	0.39
Heart failure				
Reduced ejection fraction	34 (12)	3 (8)	31 (12)	0.39
Preserved ejection fraction	44 (15)	11 (28)	33 (13)	0.02
Ischemic heart disease	103 (36)	13 (33)	90 (36)	0.75
Atrial fibrillation	95 (33)	18 (46)	77 (31)	0.06
Days admission to Holter	2 (1–4)	3 (2–4)	2 (1–4)	0.12
Abnormal baseline ECG	177 (61)	30 (77)	147 (59)	0.03
Baseline ECG abnormalities				
Any BBB^a^	65 (22)	13 (62)	52 (20)	< 0.01
Bradycardia (under 50 bpm)	40 (14)	7 (18)	33 (13)	0.42
1st or 2nd degree AVB	38 (13)	13 (33)	25 (10)	< 0.01
Atrial fibrillation/flutter	70 (24)	7 (18)	63 (25)	0.33
LVH	17 (7)	0	17 (6)	0.09
Long QT interval	14 (5)	0	14 (6)	0.13
≥ 2 ECG abnormalities	60 (21)	16 (41)	44 (18)	< 0.01
Number of abnormalities	1 (0–1)	1 (0–2)	1 (0–1)	< 0.01
Presenting condition				
Dyspnea	95 (33)	16 (41)	79 (32)	0.24
Chest pain	65 (23)	3 (8)	62 (25)	0.02
Palpitations	51 (18)	9 (23)	42 (17)	0.34
Pre-syncope	48 (17)	9 (23)	39 (16)	0.24
Systemic emboli	19 (7)	0 (0)	19 (8)	0.08
Other diagnosis	11 (4)	2 (5)	9 (4)	0.98

*ECG* electrocardiogram, *BBB* bundle branch block, *AVB* atrioventricular block, *SVT* supraventricular tachycardia, *LVH* left ventricular hypertrophy.

^a^Right, left, or bifascicular block.

### Holter outcomes

Thirty-nine of the 289 patients (13%) had a diagnostic inpatient Holter test. Palpitations and pre-syncope were the conditions with the highest diagnostic value (18% and 19%, respectively), while chest pain and systemic emboli had the lowest (5% and no diagnostic findings, respectively) (Fig. 1).

**Figure 2:**
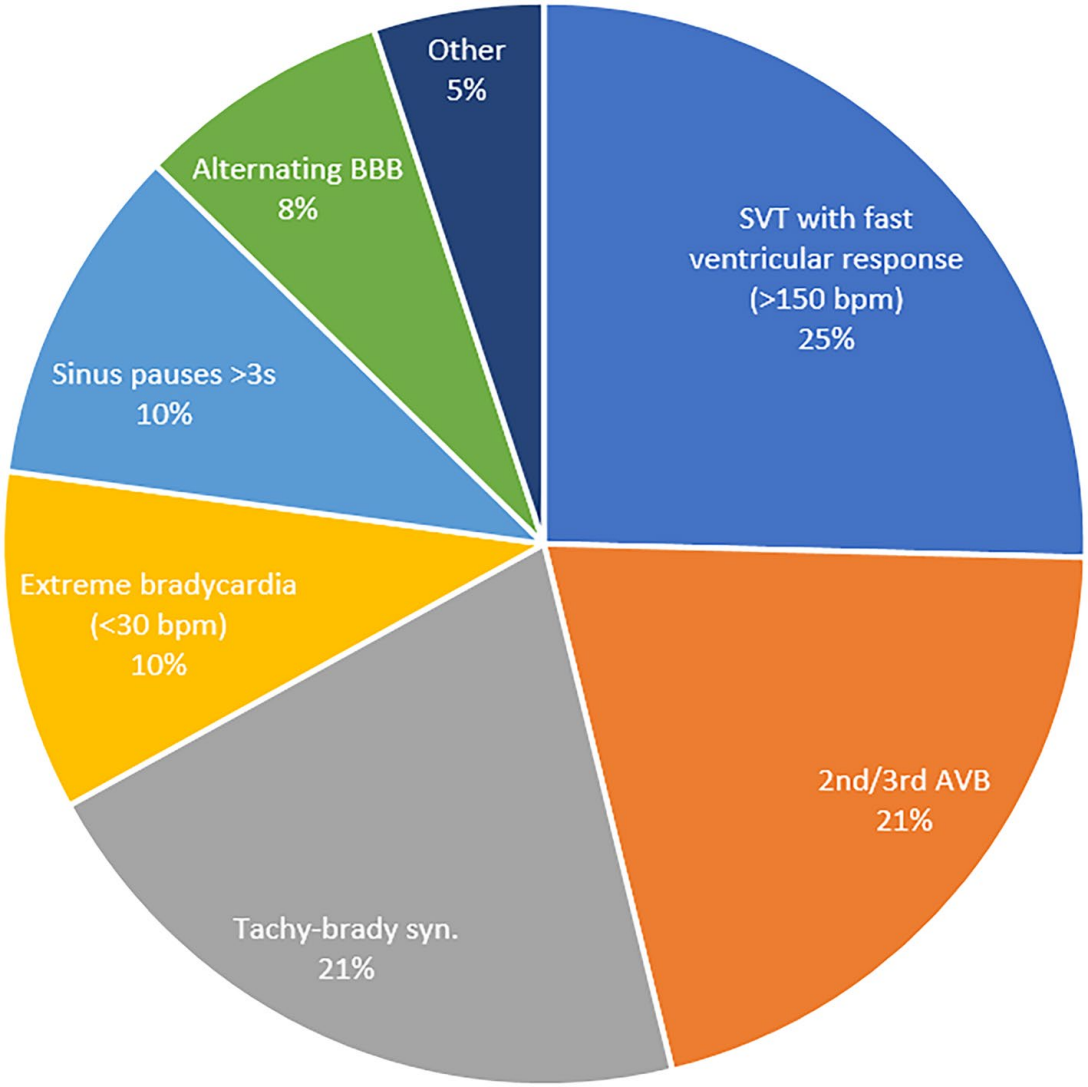
Holter diagnostic findings. *BBB* bundle branch block, *SVT* supraventricular tachycardia, *AVB* atrioventricular block, *BPM* beats per minute, Tachy-brady syn. Alternating bradycardia and atrial tachyarrhythmias.

The arrhythmias detected in the diagnostic Holter tests are presented in Fig. 2. The most frequently encountered findings were SVT with a rapid ventricular response (> 150 bpm, *n* = 10, 26%), high-degree AVB (Mobitz type 2 or 3rd degree, *n* = 8, 21%) and tachy-brady syndrome (sick-sinus syndrome, *n* = 8, 21%).

Following the diagnostic inpatient Holter results, 17 (44%) patients were referred for a pacemaker, one patient received an implantable cardioverter-defibrillator (for episodes of non-sustained VT and without), 10 (26%) initiated an anti-arrhythmic drug, 8 (21%) initiated beta blockers, and 6 (15%) stopped or had a dose adjustment of their beta blockers. Non-dihydropyridine calcium channel blocker drugs had not been previously used by any patient in the cohort and were not initiated for any of them. None of the patients experienced a cardiac arrest during the Holter exam. From the patients referred for a pacemaker, 6 (35%) had known HF (of them 5 with HFpEF), all without a prior indication for a defibrillator insertion.

Considering the utility of inpatient 24-h Holter ECG monitoring for each condition, as presented above, the added cost for every detection of an arrhythmia was as follows: $5802 USD for patients presenting with HF exacerbation or dyspnea, $21,170 USD for patients presenting with chest pain, $5536 USD for patients presenting with palpitations, and $5211 USD for patients presenting with pre-syncope.

### Predictors for a diagnostic Holter

Univariate and multivariate analysis of predictors for a diagnostic inpatient Holter ECG appear in Tables 1, 2 and 3. Hypertension (adjusted OR (AOR) 4.5, 95% CI 1.3–14.8, p = 0.02), heart failure with preserved ejection fraction (HFpEF, AOR 2.3, 95% CI 1.1–5.4, p = 0.04), ≥ 2 abnormalities in baseline ECG (AOR 2.6, 95% CI 1.2–5.7, p = 0.01) were independent predictors for diagnostic Holter ECG (Table 2). In a second multivariate regression model to evaluate the association of specific ECG findings with a diagnostic Holter (Table 3), the presence of BBB (AOR 4.2, 95% CI 1.9–9.2, p < 0.01) and AVB (first or second degree, AOR 5, 95% CI 2.04–12.3, p < 0.01) were also independent predictors.

**Table 2:** Multivariate logistic regression of predictors for diagnostic Holter monitoring.

Variable	AOR	95% CI	*p*
Age	0.99	0.97–1.03	0.88
Female sex	0.8	0.39–1.65	0.55
Hypertension	4.53	1.30–14.8	0.02
HFpEF	2.29	1.07–5.37	0.04
≥ 2 ECG abnormalities	2.65	1.23–5.71	0.01

*AOR* adjusted odds ratio, *CI* confidence interval, *HFpEF* heart failure with preserved ejection fraction, *ECG* electrocardiogram.

**Table 3:** Multivariate regression including specific ECG features as predictors for diagnostic Holter monitoring.

Variable	AOR	95% CI	*p*
Age	0.98	0.95–1.02	0.37
Female sex	0.95	0.45–2.02	0.89
Hypertension	3.85	1.07–13.8	0.04
HFpEF	2.25	0.94–5.39	0.07
Any BBB^a^	4.16	1.89–9.16	< 0.01
1st or 2nd degree AVB	5.00	2.04–12.3	< 0.01

*ECG* electrocardiogram, *AOR* adjusted odds ratio, *CI* confidence interval, *HFpEF* heart failure with preserved ejection fraction, *BBB* bundle branch block, *AVB* atrioventricular block.

^a^Right, left, or bifascicular block.

We performed a sub-analysis among patients with a diagnostic Holter result, to compare the baseline characteristics between patients with Bradycardia-related arrhythmias (sinus pauses, alternating BBB, extreme bradycardia, and high degree AVB) to tachyarrhythmia. We did not find any associations between patients' characteristics and one of the mentioned groups.

### Comparison based on Holter indications

The comparison of selected variables between the groups (based on the above predictors for diagnostic Holter) appears in Table 4. Patients in the first group (unexplained dyspnea/HF exacerbation and chest pain) had higher rates of ischemic heart disease (p < 0.001) and BBB in their baseline ECGs, while all other variables were similar between the groups. When analyzing predictors for diagnostic Holter among each group, there was no change compared to the entire cohort, except from the presence of 2 or more abnormalities in baseline ECG, that was no longer a predictor in group 2.

**Table 4:** Comparison of baseline and clinical characteristics between patients with different indications for Holter monitoring.

Variable	Group 1^a^ (n = 160)	Group 2^b^ (n = 118)	*p*
Age	76 ± 13	75 ± 15	0.47
Female sex	71 (45)	60 (52)	0.22
Hypertension	114 (72)	86 (75)	0.42
Ischemic heart disease	70 (44)	30 (25)	< 0.01
Abnormal baseline ECG	97 (61)	70 (61)	0.98
Baseline ECG abnormalities			
≥ 2 ECG abnormalities	34 (21)	23 (20)	0.78
Any BBB	44 (28)	19 (17)	0.03
1st or 2nd degree AVB	21 (13)	15 (13)	0.97
Diagnostic Holter	19 (12)	18 (16)	0.38

*ECG* electrocardiogram, *BBB* bundle branch block, *AVB* atrioventricular block.

^a^Including patients evaluated for unexplained dyspnea or chest pain.

^b^Including patients evaluated for palpitations, pre-syncope, and systemic emboli.

## Discussion

The diagnostic value of a 24-h Holter monitoring in hospital setting has not been established for conditions other than syncope or stroke. We therefore conducted this cohort study of patients who completed Holter ECG monitoring during admission to an internal medicine department with conditions other than syncope or stroke. We found five main conditions, other than syncope or stroke, that led to completion of Holter monitoring as a part of the diagnostic workup: unexplained HF/dyspnea, palpitations, chest pain, pre-syncope, and systemic emboli, all common causes for admissions^17^. The yield of inpatient Holter ECG for their evaluation has been scarcely described before. We found a varied diagnostic yield of Holter ECG for the above indications (19–0%) and estimated the cost of a single diagnostic Holter test in each of these conditions. We found independent predictors for a diagnostic Holter exam, including patients' comorbidities (hypertension and HFpEF) and baseline ECG abnormalities.

A 24-h Holter ECG monitoring is a commonly used method for monitoring inpatients as part of the evaluation of stroke or arrhythmic syncope. When telemetry monitoring is not available during hospitalization (due to a lack of telemetry beds or manpower with the appropriate skills to interpret the results in real time), Holter is often the only remaining option for inpatient monitoring. In cases of high risk for life-threatening arrhythmia or when evidence for a dangerous arrhythmia was already recoded by ECG or monitoring, telemetry is mandatory to offer immediate diagnosis and treatment. However, Holter can be a substitute in cases of lower suspicion for life-threatening arrhythmias (but still high enough suspicion of an arrhythmic cause for the patient's main reason for admission) or if the evaluation of acute conditions (such as chest pain or heart failure exacerbation) was negative for other causes but arrhythmia is still considered in the differential diagnosis after the patient's condition was stabilized. Inpatient Holter ECG could also be considered when there is a high suspicion for a non-malignant arrhythmia that could be treated before discharge (such as sinus block in pre-syncope that requires a pacemaker) or due to sociodemographic reasons that might limit patients' ability to complete this test in the ambulatory setting. Holter ECG preformed for inpatients may yield information of diagnostic value in the search for certain etiologies, consequently enabling earlier treatment. However, Holter monitoring can potentially prolong hospital stay by its lack of availability and the need for at least 24 more hours of monitoring during hospitalization, which reportedly increases the likelihood of adverse events, such as falls and acquired infections^18^. In addition, it was shown that although Holter monitoring is inexpensive in terms of set-up costs, it is expensive in terms of cost per diagnosis^19^. In recent years, inpatient Holter ECG has been shown to have a limited diagnostic yield for patients with syncope or stroke^7,9–11^. Still, restricting its use only to the ambulatory setting is not supported by hard evidence, especially for conditions other than syncope or stroke.

The presence of hypertension and more than one abnormality in the baseline ECG were found to be independent predictors for diagnostic Holter, similar to studies on patients with syncope or stroke and those describing inpatient telemetry monitoring^6,12,18,19^. On the contrary, HFpEF was not found in previous works to be associated with positive Holter test, although its association with arrhythmias is well known^20^. Baseline ECG with BBB was also found to be associated with diagnostic Holter (*P* < 0.01, Table 3). Both right and left BBB were previously shown to have prognostic implications and to be associated with other arrhythmias^14,21,22^. Of note, it seems that another important predictor for a diagnostic Holter was the original indication for choosing Holter monitoring, with a wide variation in the results between the conditions.

Holter monitoring of patients with palpitations has shown diagnostic value, with 18% diagnostic results. The yield of Holter monitoring in the evaluation of palpitations was mainly studied in the ambulatory setting and was found to have a low sensitivity (33–35%), with diagnostic findings in only 1–3% of the cases^23,24^. Sulfi et al. analyzed the results of ambulatory Holter monitoring in patients with palpitations or altered mental status and found low rates of significant arrhythmias^25^. We also found a high utility of inpatient Holter ECG among patients presenting with presyncope (19% diagnostic findings). This condition is frequently omitted from or combined with syncope datasets, and only few if any studies directly addressed its evaluation and outcomes^16,26^. Pre-syncope is a subjective and highly common condition. Grossman et al. found patients with presyncope to have similar adverse outcomes as those with syncope, while they were less likely to be admitted^27^. However, we found a higher rate of diagnostic Holter tests in patients with pre-syncope than most studies which included syncope patients^10,28^. The inpatient setting of our study might explain the difference in diagnostic rates in patients with the above conditions compared with studies performed in the ambulatory setting. We believe that admitted patients are pre-screened in the emergency department and, therefore, have a higher pre-test probability for arrhythmia that warranted their hospitalization. In other words, a correct patient selection might have led to a more limited use of inpatient Holter ECG for these highly common conditions, resulting in a high diagnostic yield.

Unexplained HF exacerbation or dyspnea (including an unexplained exacerbation of heart failure) had a 17% yield of diagnostic Holter findings. Identifying the relatively rare cases of heart failure exacerbation secondary to arrhythmia can be crucial to prevent further deterioration and recurrent hospitalizations^29^. Most dyspneic patients included in our study already had a thorough evaluation, usually including echocardiography and cardiac stress tests that were inconclusive. It is important to note that dyspnea is a highly subjective complaint among patients with cardiac and non-cardiac etiologies. Limiting the role of Holter ECG to be used only late in the evaluation of these patients could be the result of our high diagnostic yield. In addition, the use of Holter ECG for dyspnea workup is relatively rare and one can assume that there was a clue for arrhythmia in patient history or basic work-up. To the best of our knowledge, we are the first to describe the results of inpatient Holter monitoring for this indication.

Chest pain was the condition with the second lowest Holter diagnostic value (5%). A study among diabetic elderly patients found that 24-h ambulatory monitoring was able to detect ST-segment changes indicative of silent MI^30^. However, Patients with chest pain in our study did not have any evidence of acute coronary syndrome or ischemia-related changes in their Holter ECGs. Sinder et al.^31^ and Durairak et al.^32^ also found a low event rate of 0.5% and 1.4%, respectively, among patients with low-risk chest pain in a telemetry unit.

Our novel results raise questions about the contribution of Holter ECG monitoring in a non-ambulatory setting. Undoubtedly, telemetry with real-time data is needed, rather than Holter monitoring, for unstable patients and those with the risk of life-threatening arrhythmias, as mentioned above. However, for stable patients with relatively high pre-test probability (such as those with several abnormalities in their baseline ECG), and a need of a more in-depth analysis, inpatient Holter monitoring might be of some value. Using inpatient Holter ECG instead of telemetry might seems dangerous because results are only interpreted after the completion of the exam. In our cohort, none of the patients with diagnostic results required acute treatment during the Holter exam such as cardiac arrest or experienced clinical deterioration that could be prevented by using telemetry, once again stressing the need for correct patient selection. Surprisingly, while Holter monitoring is regarded as an ambulatory test for conditions other than syncope or stroke, there is only scarce data to support it. Guidelines or practice standards regarding the indications for inpatient Holter monitoring and proper patient selection, which are currently missing, can lower the extent of overuse of ECG monitoring during hospitalization, shorten its duration and set the parameters for Holter use in either inpatient or outpatient settings.

This study has some limitations. Decision to refer to Holter monitoring was made by the treating medical team based upon their assessment of risk and benefit. While that feature can lead to a selection bias, it also reflects a real-world setting. Although we included all patients in a three-year period from the nine internal medicine departments in our center, generalizability of our results should consider the single-center nature of this study. Given the retrospective design of our study, comorbidities that were not documented or treated might have been missed at data collection. To divide our study population based on their main condition that indicated a Holter monitoring, we reviewed the main complaints and reasons for Holter monitoring. Patients with a similar indication for Holter ECG have different baseline and clinical characteristics. As such, the decision to refer to Holter monitoring in the hospital setting should be made per patient and not per condition. Finally, while a negative Holter result can add value in terms of excluding an arrhythmia, it did not affect the treatment in any of our patients, and therefore were regarded as non-diagnostic.

In conclusion, the presented findings contribute to establishing the diagnostic value of inpatient 24-h Holter monitoring for a variety of conditions. Considering our results, the inpatient setting should not be considered as a "contraindication" for the use of Holter ECG, especially with the correct patient selection. Patient's comorbidities and baseline ECG findings emerged as the strongest factors to consider when deciding whether to refer to Holter monitoring. Further studies targeting specific populations with comparison to other monitoring methods such as loop recorder or intrathoracic cardiac monitoring, could contribute for drafting new guidelines for inpatient Holter ECG monitoring.

## Data Availability

All data generated or analyzed during this study are included in this article. Due to ethical and privacy concerns the primary dataset cannot be made openly available. The study was done retrospectively and according to the regulations of our institution review board such data could be openly shared. Request for the dataset supporting our results can be made via helsinki@tlvmc.gov.il and will be given by the first author after approval.
